# Suppression of esophageal tumor growth and chemoresistance by directly targeting the PI3K/AKT pathway

**DOI:** 10.18632/oncotarget.2596

**Published:** 2014-10-18

**Authors:** Bin Li, Jin Li, Wen Wen Xu, Xin Yuan Guan, Yan Ru Qin, Li Yi Zhang, Simon Law, Sai Wah Tsao, Annie L.M. Cheung

**Affiliations:** ^1^ Department of Anatomy, Li Ka Shing Faculty of Medicine, The University of Hong Kong, Pokfulam, Hong Kong SAR, China; ^2^ Centre for Cancer Research, Li Ka Shing Faculty of Medicine, The University of Hong Kong, Pokfulam, Hong Kong SAR, China; ^3^ The University of Hong Kong-Shenzhen Institute of Research and Innovation (HKU-SIRI), China; ^4^ Department of Clinical Oncology, Li Ka Shing Faculty of Medicine, The University of Hong Kong, Pokfulam, Hong Kong SAR, China; ^5^ Department of Clinical Oncology, First Affiliated Hospital, Zhengzhou University, Zhengzhou, China; ^6^ Department of Surgery, Li Ka Shing Faculty of Medicine, The University of Hong Kong, Pokfulam, Hong Kong SAR, China; ^7^ Present Address: Research Center for Molecular Medicine of The Austrian Academy of Sciences, Vienna, Austria

**Keywords:** PI3K/AKT, targeted therapy, esophageal cancer, tumor growth, chemoresistance

## Abstract

Esophageal cancer is the sixth most common cause of cancer-related deaths worldwide. Novel therapeutic intervention is urgently needed for this deadly disease. The functional role of PI3K/AKT pathway in esophageal cancer is little known. In this study, our results from 49 pairs of human esophageal tumor and normal specimens demonstrated that AKT was constitutively active in the majority (75.5%) of esophageal tumors compared with corresponding normal tissues. Inhibition of the PI3K/AKT pathway with specific inhibitors, wortmannin and LY294002, significantly reduced Bcl-xL expression, induced caspase-3-dependent apoptosis, and repressed cell proliferation and tumor growth *in vitro* and *in vivo* without obvious toxic effects. Moreover, significantly higher expression level of p-AKT was observed in fluorouracil (5-FU)-resistant esophageal cancer cells. Inactivation of PI3K/AKT pathway markedly increased the sensitivity and even reversed acquired resistance of esophageal cancer cells to chemotherapeutic drugs *in vitro*. More importantly, the resistance of tumor xenografts derived from esophageal cancer cells with acquired 5-FU resistance to chemotherapeutic drugs was significantly abrogated by wortmannin treatment in animals. In summary, our data support PI3K/AKT as a valid therapeutic target and strongly suggest that PI3K/AKT inhibitors used in conjunction with conventional chemotherapy may be a potentially useful therapeutic strategy in treating esophageal cancer patients.

## INTRODUCTION

Esophageal cancer is the 8^th^ most common cancer in the world. Although the incidence rate of esophageal cancer pales in comparison with that of cancers of breast, prostate, colon, and lung, it has a very high lethality rate, with more than 400,000 deaths (i.e. around 90% of the incidence of the disease) reported in 2012 [1, 2]. The 5-year survival rate of patients with esophageal cancer rarely exceeds 40% [3]. Local recurrence after initial treatment is still the major cause of treatment failure in the patients [4, 5]. Thus, a detailed study of esophageal cancer including identification and better understanding of the key signaling pathways responsible for development and progression of this disease is urgently needed to develop new treatment strategies.

Genetic abnormalities of the phosphatidylinositil-3- kinase (PI3K)/AKT pathway are common in human cancer, and there is increasing evidence of PI3K/AKT being involved in the development of many types of cancers [6, 7], thus making PI3K/AKT and its downstream pathways promising targets for therapeutic intervention [8, 9]. However, the role of PI3K/AKT signaling pathway in esophageal tumorigenesis is not fully understood and the feasibility of targeting PI3K/AKT as a potential treatment for esophageal cancer has not been elucidated. Recent immunohistochemical studies including ours showed that PI3K/AKT is constitutively activated in human esophageal tumor tissues [10], but studies that evaluate PI3K/AKT signaling based on matched tumor and normal tissues are limited. More importantly, there is currently very little *in vitro* or *in vivo* experimental data on the effects of PI3K/AKT inhibition on esophageal cancer.

Intrinsic and acquired resistance to chemotherapeutic drugs in human cancer may lead to poor treatment response or cancer recurrence. Fluorouracil (5-FU) is a key chemotherapy drug for esophageal cancer. We recently established 5-FU-resistant (FR) cell lines by treating esophageal cancer cells with increasing concentration of 5-FU for over one year [11], and here we observed significantly increased expression of phosphorylated-AKT (p-AKT), the activated form of AKT, in the FR cells. In the present study, we aim to demonstrate, the significance of PI3K/AKT activation in esophageal cancer by examining the p-AKT expression in paired clinical tumor and normal specimens, and to determine the effects of specific inhibitors of PI3K/AKT on caspase-3-dependent cancer cell apoptosis, esophageal tumor growth and chemoresistance by *in vitro* experiments and *in vivo* tumorigenesis model.

## RESULTS

### PI3K/AKT pathway is constitutively activated in esophageal tumors compared with paired normal tissues

**Figure 1:**
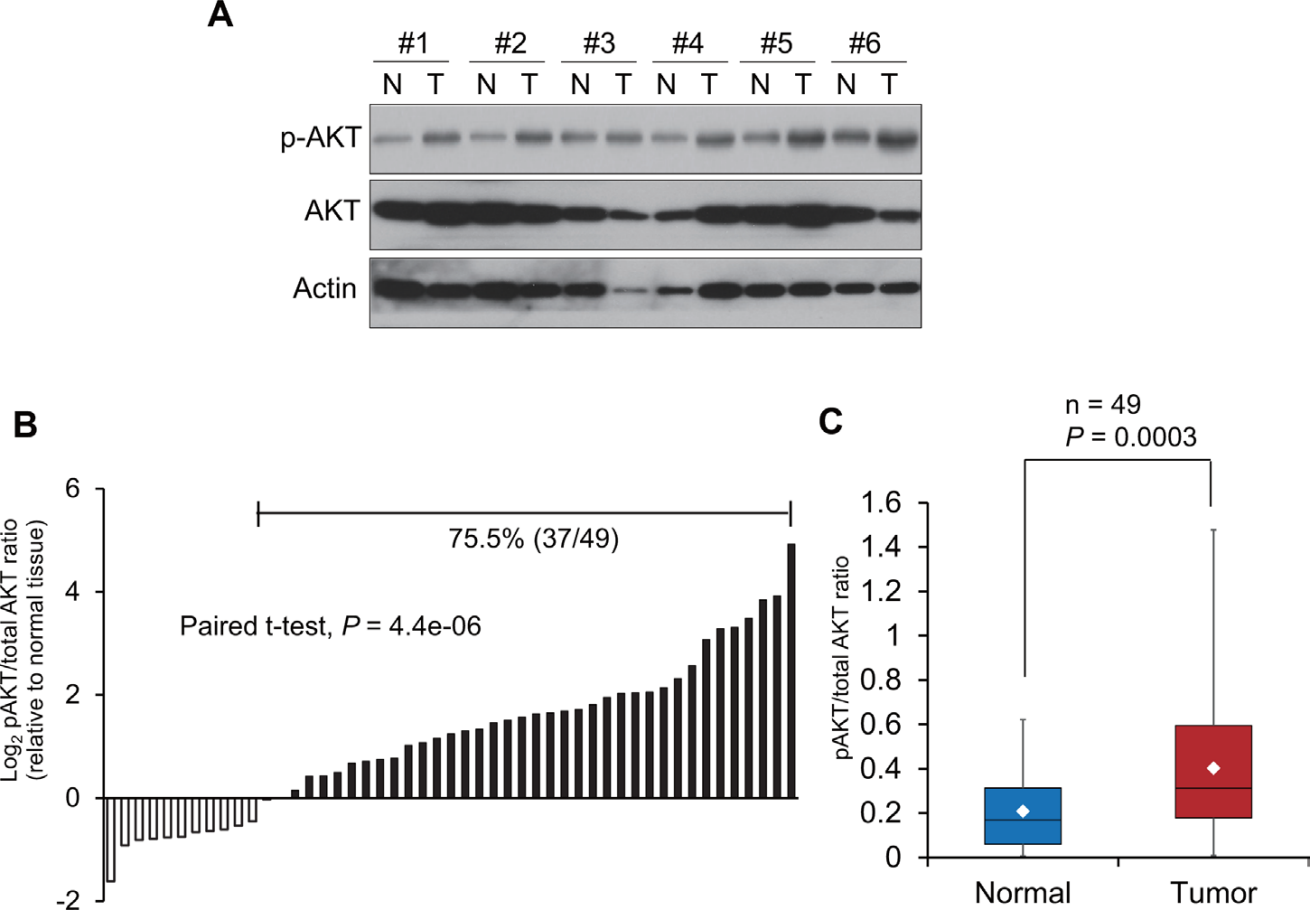
Constitutive activation of PI3K/AKT signaling pathway in esophageal cancer (A) Expression levels of p-AKT and total AKT were determined in 49 pairs of esophageal tumor and matched normal tissues by Western blot, and results of 6 representative esophageal tumor tissues (T) and their matched normal tissues (N) were shown. Actin was included as loading control. (B) p-AKT/total AKT ratio in 49 tumor tissues relative to matched normal esophageal tissues. Higher ratio of p-AKT to total AKT was found in 75.5% (37 of 49) of human primary esophageal cancer, compared with their corresponding normal tissues. (C) Comparison of p-AKT/total AKT ratios between tumor tissues and normal tissues. The boxes contain the values between 25^th^ and 75^th^ percentiles of the 49 cases, and the whiskers extend to the highest and lowest values. The lines across the boxes indicate the median values, and the white diamonds inside the boxes represent the mean values.

To study whether the PI3K/AKT signaling pathway is clinically relevant in esophageal cancer, the expression levels of p-AKT and total AKT were determined in 49 pairs of human esophageal tumor and adjacent normal tissues (Figure 1A). Compared with the corresponding normal tissues, a higher p-AKT/total AKT ratio was observed in the majority of primary esophageal tumors studied (37 of 49; 75.5%) (Figure 1B). As seen in Figure 1C, the mean p-AKT/total AKT ratio in the tumor tissues was about 2-fold higher than that in the paired normal tissues (0.40 ± 0.32 versus 0.21 ± 0.17; *P* < 0.001). These data highlighted the clinical relevance of the PI3K/AKT pathway and its potential as therapeutic target in esophageal cancer.

### PI3K/AKT inhibition decreases Bcl-xL expression and induces apoptosis in esophageal cancer cells

**Figure 2:**
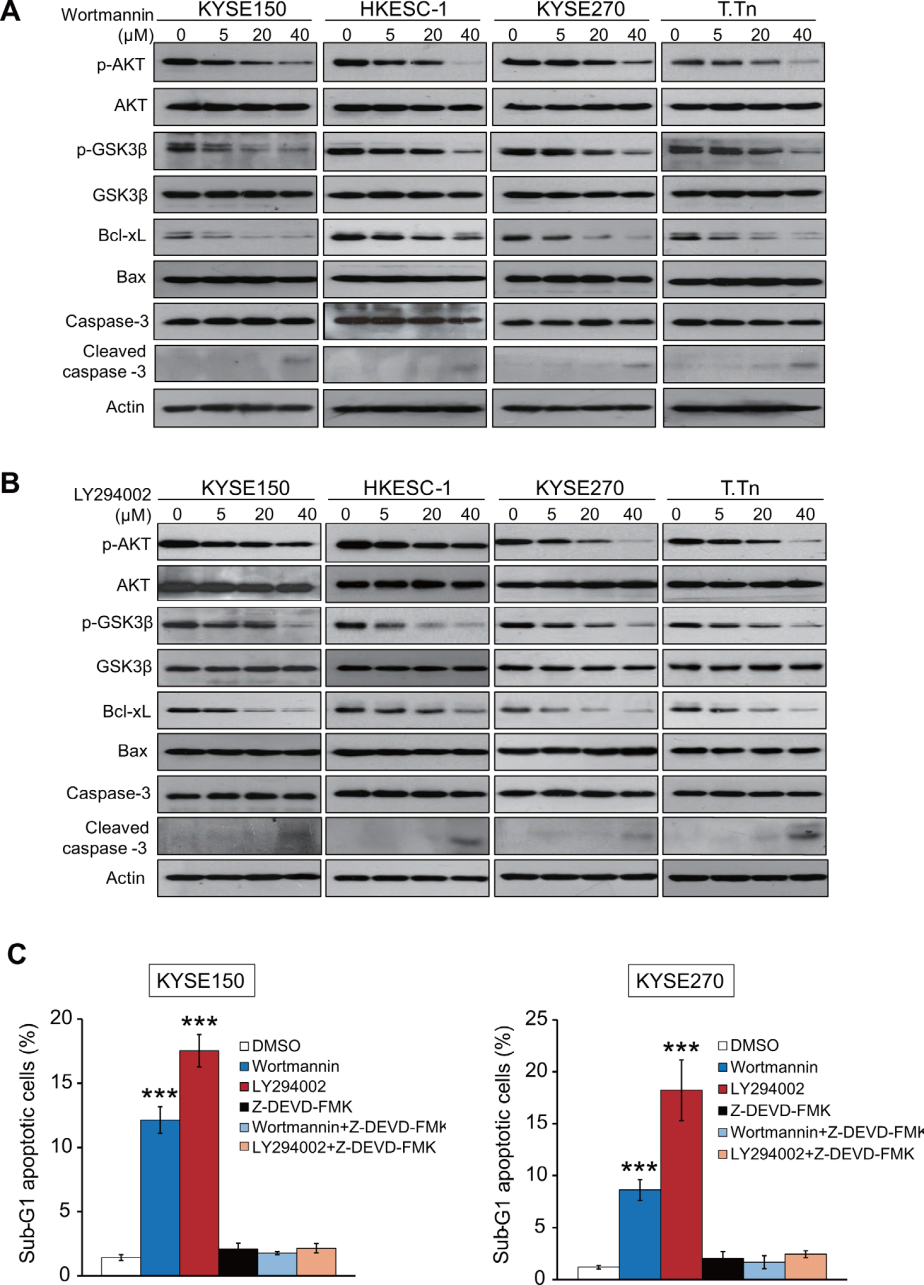
Effects of wortmannin and LY294002 on PI3K/AKT pathway and expressions of apoptosis-associated proteins Four esophageal cancer cell lines were treated with different concentrations of wortmannin (A) or LY294002 (B) respectively for 48 h, and cell lysates were collected for Western blot analysis of p-AKT, AKT, p-GSK3β, GSK3β, Bcl-xL, Bax, caspase-3, and cleaved caspase-3. (C) Comparison of sub-G1 population percentage by flow cytometry in the esophageal cancer cells treated with wortmannin (40 μM), LY294002 (40 μM), Z-DEVD-FMK (50 μM) alone, or a combination of Z-DEVD-FMK and wortmannin or LY294002. Bars, SD; *** *P* < 0.001 compared with DMSO-treated cells.

Two specific inhibitors, wortmannin and LY294002, were used in this study to block the PI3K/AKT signaling pathway. As shown in Figure 2A, treatment with wortmannin resulted in a dose-dependent reduced phosphorylation of AKT (p-AKT) and its downstream target GSK3β (p-GSK3β), but not total AKT or GSK3β, in four esophageal cancer cell lines, KYSE150, HKESC-1, KYSE270, and T.Tn. In addition, decreased Bcl-xL and increased cleaved caspase-3 expressions were detected upon treatment, although the expression level of Bax and caspase-3 remained stable (Figure 2A). These experiments were repeated with LY294002 in the four cell lines and similar results were obtained (Figure 2B). We also found that wortmannin and LY294002 significantly increased the percentage of sub-G1 esophageal cancer cell population, whereas addition of Z-DEVD-FMK, a caspase-3 inhibitor, markedly abrogated these effects (Figure 2C). These data indicated that wortmannin and LY294002 exerted dose-dependent inhibitory effects on the PI3K/AKT pathway and pro-apoptotic proteins, therefore inducing caspase-3-dependent apoptosis in esophageal cancer cells.

### Wortmannin and LY294002 reduce proliferation and colony-formation ability of esophageal cancer cells

**Figure 3:**
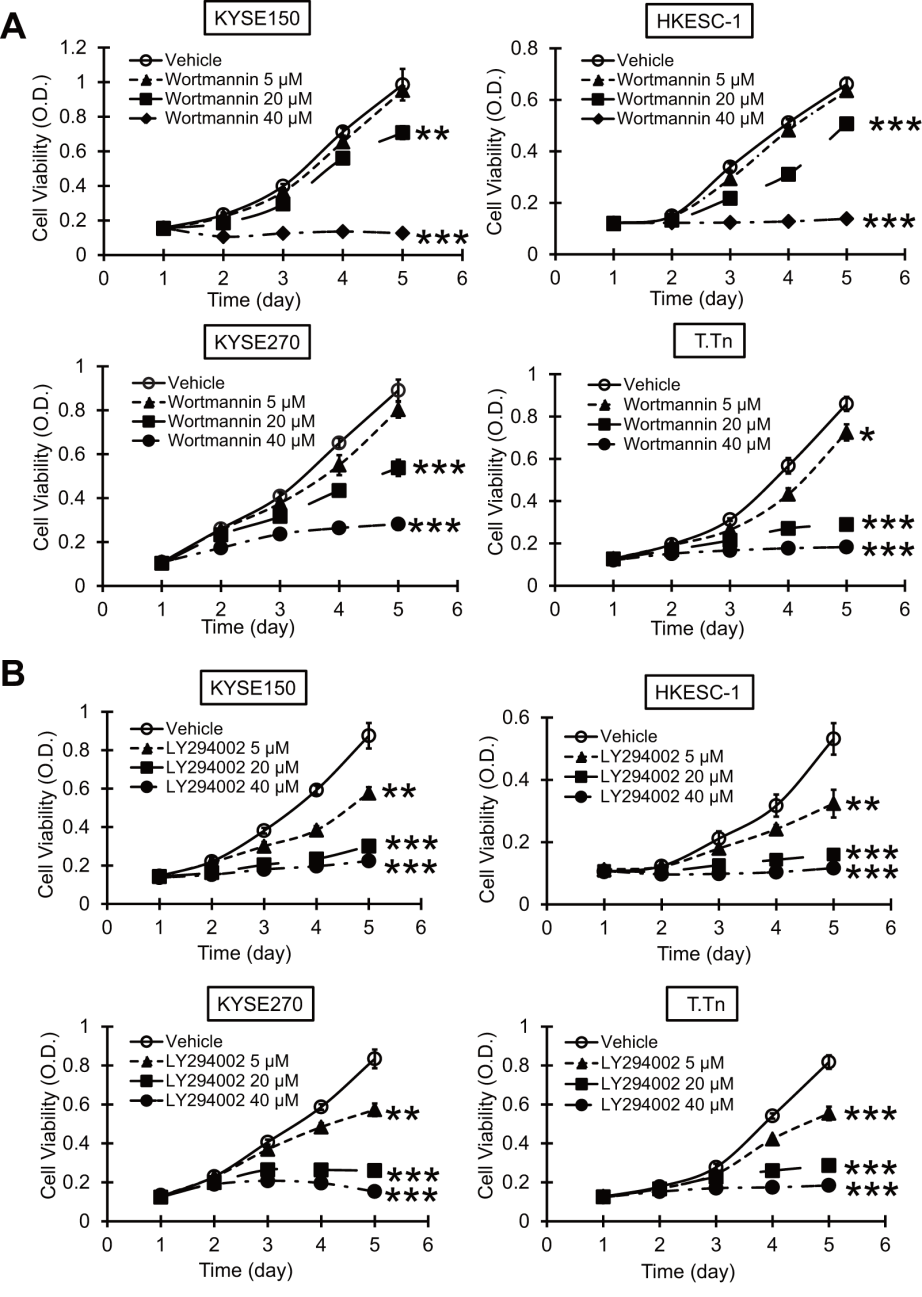
Effects of PI3K/AKT inhibitors on esophageal cancer cell proliferation MTT assay was used to determine the effects of different concentrations of wortmannin (A) and LY294002 (B) on viability of esophageal cancer cells lines KYSE150, HKESC-1, KYSE270, and T.Tn. Bars, SD; * *P* < 0.05; **, *P* < 0.01; ***, *P* < 0.001 compared with DMSO-treated cells.

**Figure 4:**
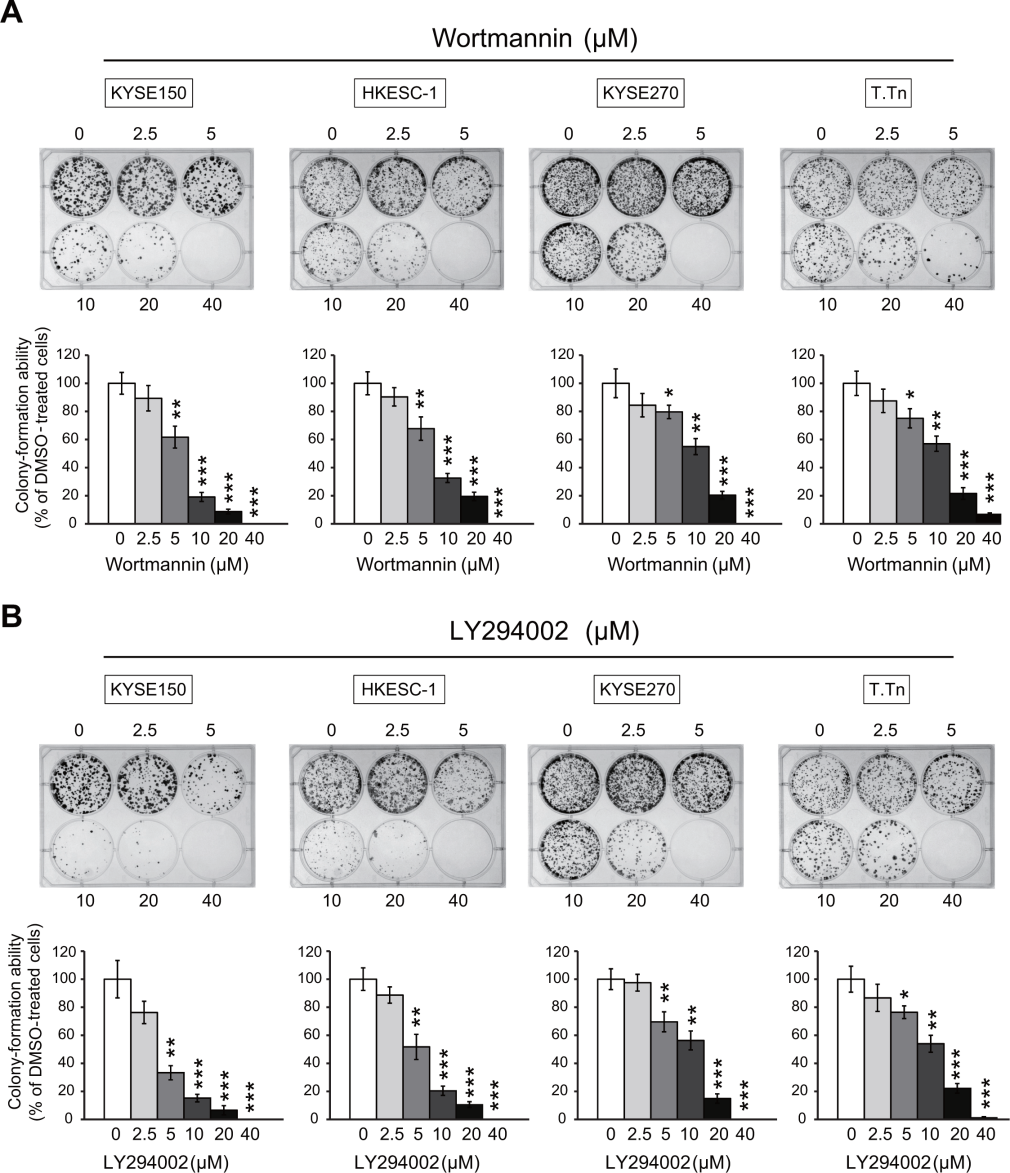
Effects of PI3K/AKT inhibitors on colony-formation ability of esophageal cancer cells Colony-formation assay showed that exposure of the four esophageal cancer cell lines to wortmannin (A) and LY294002 (B) decreased colony-formation ability in a dose-dependent manner. Bars, SD; * *P* < 0.05; **, *P* < 0.01; ***, *P* < 0.001 compared with DMSO-treated cells.

The esophageal cancer cell lines KYSE150, HKESC-1, KYSE270, and T.Tn were exposed to different concentrations of wortmannin or LY294002, and the results from MTT and colony-formation assays showed that blockade of PI3K/AKT pathway inhibited proliferation (Figure 3) and colony-formation ability (Figure 4) of esophageal cancer cells, suggesting that specific inhibitors of PI3K may have antitumor effects.

### Wortmannin suppresses growth of human esophageal cancer xenografts in nude mice

**Figure 5:**
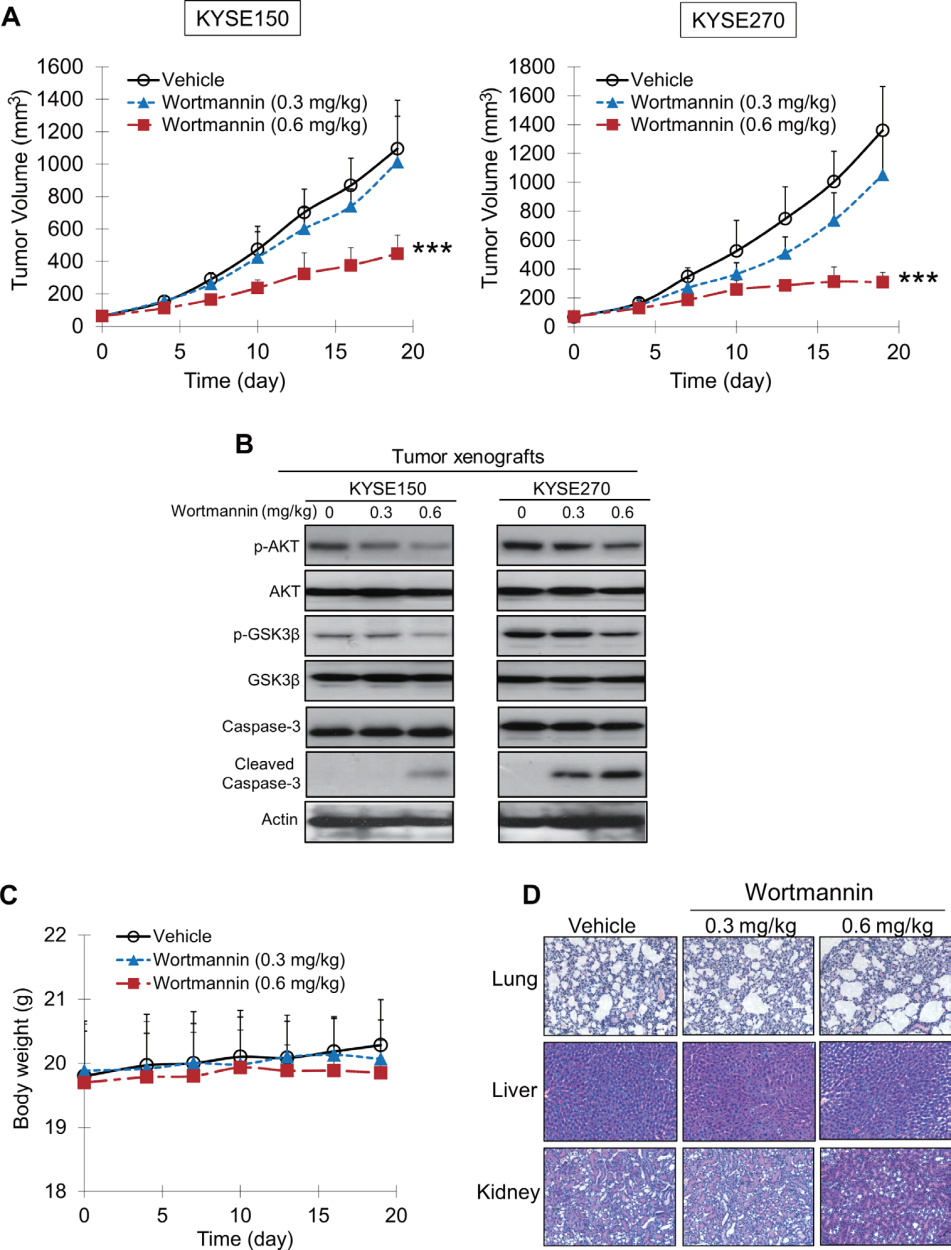
Effects of wortmannin on suppressing growth of human esophageal tumor xenografts in nude mice (A) Human esophageal cancer cells KYSE150 (left panel) and KYSE270 (right panel) were injected subcutaneously into the flanks of nude mice (n = 6 per group). Treatment of the mice with 0.6 mg/kg wortmannin twice a week significantly delayed the growth of the tumor xenografts. Note that the treatment had a better response in KSYE270-derived xenografts. (B) Western blot analysis indicated reduced expression levels of p-AKT, p-GSK3β, and increased cleaved caspase-3 in the tumors of wortmannin-treated mice, compared with the DMSO-treated group. (C, D) Comparison of body weight (C) and histological examination of lung, liver and kidney specimens (D) between wortmannin-treated and vehicle-treated animals showed no toxic effects. Bars, SD; *** *P* < 0.001 compared with DMSO-treated mice.

Nude mice bearing human esophageal tumor xenografts were used to test the therapeutic potential of wortmannin *in vivo*. As shown in Figure 5A, treatment with wortmannin for 19 days caused a significant dose-dependent suppression of tumor volume, with decreases of 59.2% and 77.3% for KYSE150 and KYSE270 tumors, respectively, in the groups receiving 0.6 mg/kg wortmannin treatment. Western blot analysis of tumor xenografts showed that wortmannin treatment resulted in inactivation of PI3K/AKT pathway and induction of apoptosis, as indicated by the decreased expression levels of p-AKT and p-GSK3β, as well as increased cleaved caspase-3. Notably, wortmannin treatment had a better response in suppressing growth of KYSE270 tumor xenografts, which expressed higher level of p-AKT (Figure 5B). There was no significant difference between the treated and control groups in terms of body weight (Figure 5C) and morphology of lung, liver, and kidneys (Figure 5D), suggesting no obvious adverse effects on animals.

### PI3K/AKT inhibition enhances the sensitivity of esophageal cancer cells to chemotherapeutic drugs *in vitro* and *in vivo*

**Figure 6:**
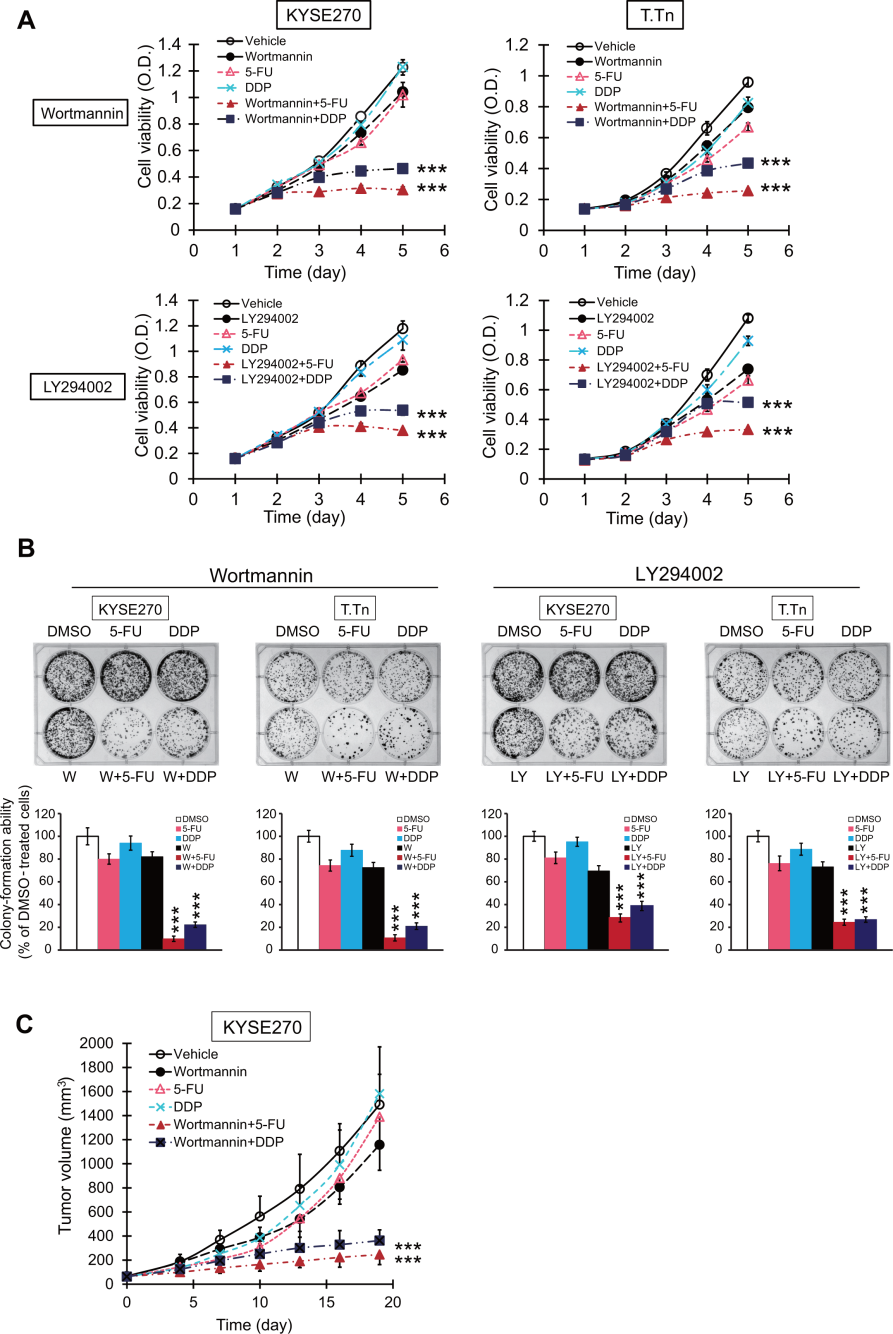
Effects of PI3K/AKT inhibitors on sensitivity of esophageal cancer cells to conventional chemotherapeutic drug *in vitro* and *in vivo* (A) Esophageal cancer cells were treated with wortmannin (5 μM), LY294002 (5 μM), 5-FU (2.5 μM), DDP (10 μM), wortmannin plus 5-FU, wortmannin plus DDP, LY294002 plus 5-FU, or LY294002 plus DDP, and cell viability and colony-formation ability measured by MTT (A) and colony-formation assays (B). (C) Nude mice xenografted with human esophageal cancer cells KYSE270 were treated with wortmannin (0.3 mg/kg), 5-FU (20 mg/kg), DDP (2 mg/kg), a combination of wortmannin and 5-FU, or a combination of wortmannin and DDP twice weekly (n = 6 per group). Bars, SD; *** *P* < 0.001 compared with 5-FU- or DDP-treated cells or mice.

The effects of PI3K/AKT inhibitors on chemoresistance were studied. The MTT (Figure 6A) and colony-formation assays (Figure 6B) data showed that PI3K/AKT inhibition significantly increased the sensitivity of esophageal cancer cell lines to 5-FU and cisplatin (DDP). The effect of wortmannin in repressing chemoresistance was also investigated *in vivo*. Nude mice bearing KYSE270 tumor xenografts were treated with 5-FU and cisplatin, with or without wortmannin. The results demonstrated that wortmannin treatment significantly enhanced the sensitivity of the esophageal tumor xenografts to chemotherapeutic drugs in animals (Figure 6C).

### PI3K/AKT inhibition reverts acquired chemoresistance of 5-FU-resistant (FR) esophageal cancer cells to chemotherapeutic drugs *in vitro* and *in vivo*

**Figure 7:**
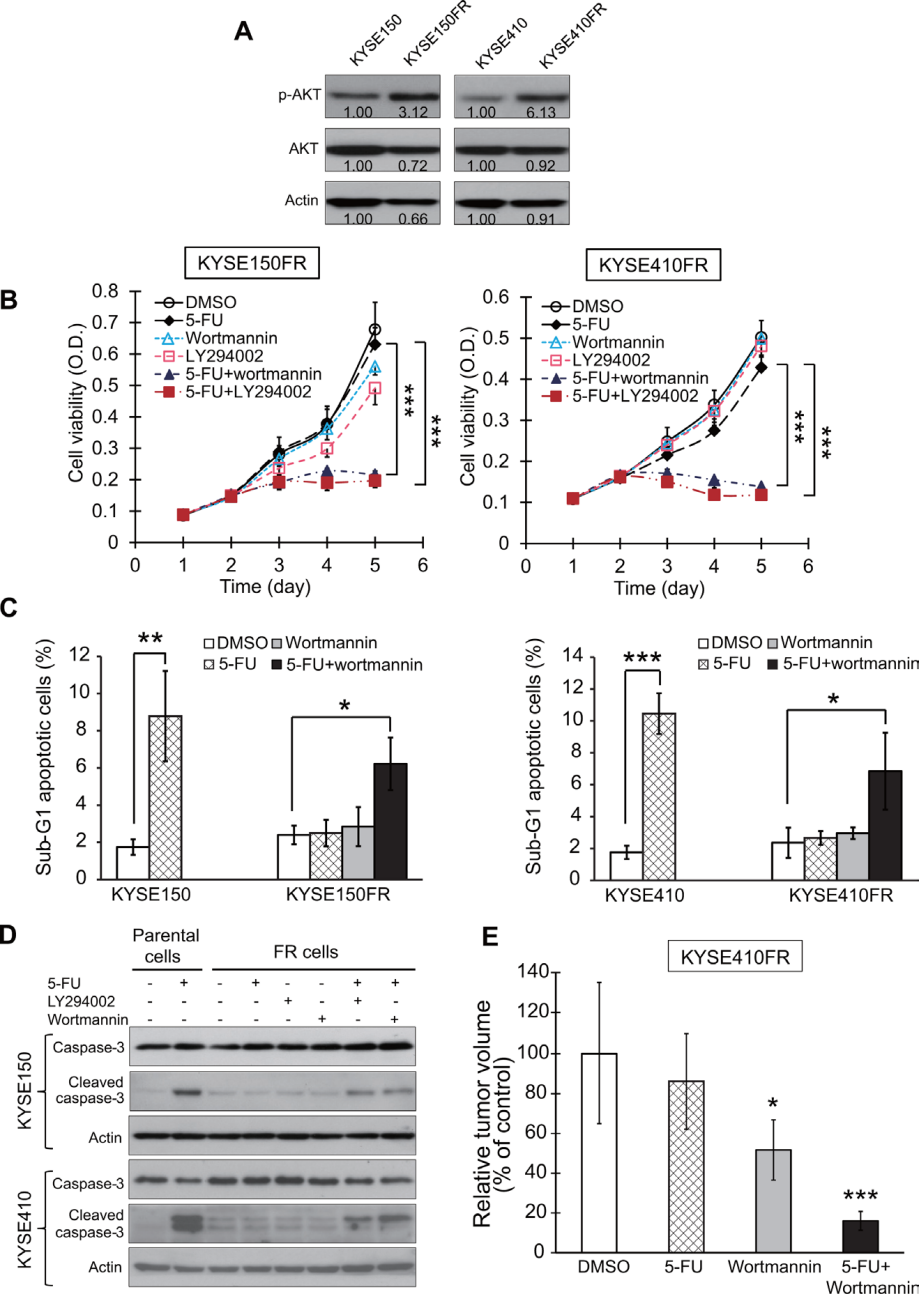
Effects of PI3K/AKT inhibition on reversing resistance of FR cells to 5-FU (A) Western blot analysis of expression levels of p-AKT and AKT in FR and corresponding parental cells. The bands were quantified using ImageJ. (B) Comparison of the viability of FR esophageal cancer cells treated with 5-FU (40 μM for KYSE150FR and 10 μM for KYSE410FR), wortmannin (5 μM), LY294002 (5 μM) alone, or a combination of 5-FU and wortmannin or LY294002. (C-D) Parental KYSE150 and KYSE410 cells were treated with 5-FU, whereas the respective FR cells were treated with 5-FU, wortmannin, LY294002 alone, or the combination of 5-FU and wortmannin or LY294002 for 48 h. The drugs were used at the same concentrations as in (B). The cells were collected for cell cycle analysis of sub-G1 population percentage using flow cytometry (C), and for Western blot analysis of caspase-3 and cleaved caspase-3 (D), respectively. (E) Nude mice bearing KYSE410FR-derived tumor xenografts were treated with wortmannin (0.3 mg/kg), 5-FU (20 mg/kg), or a combination of wortmannin and 5-FU twice weekly for three weeks (n = 6 per group). Bars, SD; * *P* < 0.05; **, *P* < 0.01; ***, *P* < 0.001.

We then proceeded to investigate the role of PI3K/AKT pathway in acquired chemoresistance. Interestingly, increased activation of PI3K/AKT pathway, indicated by higher p-AKT expression, was observed in the FR cells compared with corresponding parental cells (Figure 7A), suggesting the significance of PI3K/AKT in acquired chemoresistance in esophageal cancer cells. This prompted us to explore whether PI3K/AKT inhibitors can inhibit the proliferation of FR esophageal cancer cells in 5-FU. The results showed that although 5-FU, wortmannin, or LY294002 alone at the dosages used had no or only slight effects on the proliferation of KYSE150FR and KYSE410FR cells, combining 5-FU with wortmannin or LY294002 significantly reverted the resistance and lowered the proliferation rate (Figure 7B). Furthermore, the FR cell lines which were resistant to 5-FU-induced apoptosis were rendered apoptotic by the addition of wortmannin or LY294002, as evidenced by the increase in sub-G1 population and cleaved caspase-3 expression (Figure 7C-D). More importantly, our *in vivo* experiments showed that the resistance of KYSE410FR tumor xenografts to 5-FU treatment in nude mice was markedly abrogated by wortmannin treatment (Figure 7E). Taken together, the above findings indicated that blockade of PI3K/AKT can reverse acquired resistance of FR esophageal cancer cells to chemotherapy drugs.

## DISCUSSION

PI3K is a lipid kinase that generates second messenger lipid phosphatidylinositol (3-5)-triphosphate (PIP3), which recruits and activates a number of proteins including AKT. AKT encodes a serine/threonine kinase, and is activated through phosphorylation, which mediates the activation of target genes, therefore regulating cell proliferation, survival, angiogenesis, invasion and metastasis. Constitutively activated PI3K/AKT pathway is a common event in many types of cancer, and is associated with poor prognosis and reduced survival [6, 7, 12]. In esophageal cancer, the association of genetic variants of AKT with chemoradiotherapy response and survival has been reported [13]. Our previous studies demonstrated constitutive activation of the PI3K/AKT pathway in esophageal tumor tissues and its regulation by Id1 (inhibitor of differentiation or DNA binding) [10, 14]. In this study, Western blot analysis of clinical esophageal tumor specimens demonstrated markedly higher p-AKT expression in tumor tissues compared with paired normal tissues.

The significance of PI3K/AKT and its potential as a therapeutic target for cancer treatment have been investigated in several types of human cancer in preclinical studies, including renal cancer [15], lung cancer [16], breast cancer [17], glioblastoma [18], and neuroblastoma [19]. More importantly, the data from phase I clinical trial suggested that treatment of the cancer patients with PI3K inhibitors was associated with prolonged stable disease, and phase II trials are in progress [20, 21]. Although overexpression of PTEN (phosphatase and tensin homolog), which acts as a lipid phosphatase to dephosphorylates PIP3 and therefore negatively regulates PI3K/AKT pathway, was found to suppress esophageal cancer cell growth [22], experimental data on direct targeting of PI3K/AKT in the preclinical setting are limited. Our *in vitro* and *in vivo* results showed that specific inhibitors of PI3K/AKT inhibited proliferation and promoted apoptosis of esophageal cancer cells in a dose-dependent manner (Figures 2-5), thus indicating the therapeutic potential of targeting PI3K/AKT in esophageal cancer treatment.

Chemotherapy is now widely used in clinical cancer treatment but development of chemoresistance can compromise treatment or even result in recurrence of the disease. The contribution of chemoresistance to low survival rate of human cancer patients has therefore prompted studies that explore the underlying mechanisms. Here, we found increased constitutive activity of the PI3K/AKT signaling pathway in esophageal cancer cells with acquired resistance to 5-FU. Several *in vitro* studies have shown that inhibition of PI3K/AKT pathway can sensitize a variety of other types of cancer cells to chemotherapeutic drugs [23, 24], but the effects of specific inhibitors of PI3K/AKT on esophageal cancer chemoresistance have not been reported. Our current findings showed for the first time that wortmannin and LY294002 significantly enhanced the sensitivity of parental esophageal cancer cells and chemoresistant sublines to chemotherapeutic drugs not only *in vitro*, but also in xenografted animal models (Figures 6-7), which strongly suggest that combining PI3K/AKT inhibitors and conventional chemotherapeutic drugs may be a potentially useful therapeutic strategy in treating esophageal cancer patients, particularly as upfront therapy before tumors have a chance to develop chemoresistance. This notion is further corroborated by Yoshioka *et al*.'s multivariate analysis of paired esophageal tumor samples obtained before and after chemotherapy, which shows that p-AKT expression is the only independent predictor of poor prognosis in esophageal cancer patients with chemotherapy [25].

Taken together, we have demonstrated the significance of PI3K/AKT pathway in malignant progression of esophageal cancer, and have provided the first evidence that PI3K/AKT inhibitors can increase the sensitivity and even reverse acquired resistance of esophageal cancer cells to chemotherapeutic drugs. Genetic mutations in the PI3K/AKT pathway are common in human cancer [26]. A recent study showed that patients with gynecologic and breast cancer that have *PIK3CA* mutations are more responsive to treatment with PI3K/AKT/mTOR inhibitors than patients without mutations [21]. The effects of PI3K/AKT inhibitors on esophageal cancer with genetic variations in the PI3K/AKT pathway warrant further investigation.

## MATERIALS AND METHODS

### Cell culture and drugs

Human esophageal cancer cell lines KYSE150, KYSE270 [27], HKESC-1 [28], T.Tn [29], and the 5-FU-resistant cell lines (KYSE150FR, KYSE410FR) we established previously [11] were maintained in RPMI 1640 (Sigma, St. Louis, MO) supplemented with 10% fetal bovine serum (Invitrogen, Gaithersburg, MD) at 37 ºC in 5% CO2. Wortmannin, LY294002, 5-FU, cisplatin purchased from Calbiochem (Darmstadt, Germany), and Z-DEVD-FMK (Cayman Chemical, Ann Arbor, MI) were dissolved in DMSO.

### Esophageal cancer patient tissue specimens

Fresh human esophageal tumor and the corresponding adjacent non-neoplastic esophageal tissues were collected with informed consent and Institutional Review Board approval from 49 patients undergoing surgical resection of primary esophageal tumor at Queen Mary Hospital in Hong Kong from 2011 to 2014, and at the First Affiliated Hospital, Zhengzhou University in Zhengzhou from 2008 to 2010. All specimens were snap-frozen immediately in liquid nitrogen and stored at −80ºC.

### Western blot

Preparation of cell and tumor lysates, and details of immunoblotting were described previously [14]. The primary antibodies used included phospho-AKT (Ser473), AKT, phospho-GSK3 (Ser9), GSK3β, Bcl-xL, Bax, caspase-3 and cleaved caspase-3 obtained from Cell Signaling Technology (Beverly, MA), and actin from Santa Cruz Biotechnology (Santa Cruz, CA).

### 3-(4,5-Dimethyl thiazol-2-yl)-2,5-diphenyl tetrazolium bromide (MTT) assay

Cell viability was measured using MTT assay [30]. The absorbance was measured at a wavelength of 570 nm on a Labsystems Multiskan microplate reader (Merck Eurolab, Dietikon, Switzerland).

### Colony-formation assay

Colony-formation assay was carried out as described previously [30]. Briefly, about 500 cells were seeded per well in 6-well-plates 24 h before the addition of drugs. After 14 days, the cells were fixed in 75% ethanol and stained with 0.2% crystal violet. The numbers of colonies were counted using QuantityOne software (Bio-Rad, Hercules, CA).

### Flow cytometric cell cycle analysis

Esophageal cancer cells were harvested and washed in PBS, then fixed in 70% ethanol. Cells were washed and incubated in propidium iodide (PI) staining buffer (PBS containing 40 μg/ml PI and 40 μg/ml RNase) at 37 ºC for 30 min. The sub-G1 percentage of samples was determined on a BD FACSCanto II Analyzer (BD Biosciences, San Jose, CA) and data analyzed using FlowJo software (Tree Star Inc., Ashland, OR).

### Tumorigenicity in nude mice

Female BALB/c nude mice aged 6-8 weeks were maintained under standard conditions and cared for according to the institutional guidelines for animal care. The esophageal cancer cells suspended in a 1:1 mixture of PBS/Matrigel were subcutaneously injected into the flank of mice. The mice were randomized into treatment and control groups when the tumors reached ~5 mm diameter. The treatment groups received wortmannin (0.3 mg/kg or 0.6 mg/kg), 5-FU (20 mg/kg) or cisplatin (2 mg/kg) through intraperitoneal injection twice weekly, whereas the control groups received the vehicle only. Additional treatment groups were given wortmannin (0.3 mg/kg) combined with 5-FU (20 mg/kg) or cisplatin (2 mg/kg). The body weight of mice was monitored weekly during the experiments to evaluate overall health. Tumor size was measured with calipers every three days and tumor volume was calculated using the equation V= (length x width^2^) /2. Tumors, together with pieces of liver, lung, and kidney, were collected at the end of experiments for further analyses. All the animal experiments were approved by the Committee on the Use of Live Animals in Teaching and Research of the University of Hong Kong.

### Statistical analysis

The data were expressed as the mean ± SD and compared using ANOVA. *P* values < 0.05 were deemed significant. All *in vitro* experiments were repeated at least three times.

## References

[1] Bray F, Ren JS, Masuyer E, Ferlay J (2013). Global estimates of cancer prevalence for 27 sites in the adult population in 2008. Int J Cancer.

[2] Ferlay J, Soerjomataram I, Ervik M, Dikshit R, Eser S, Mathers C, Rebelo M, Parkin DM, Forman D, Bray F (2013). GLOBOCAN 2012 v1.0, Cancer Incidence and Mortality Worldwide: IARC CancerBase No. 11 [Internet].

[3] Kelsen DP, Ginsberg R, Pajak TF, Sheahan DG, Gunderson L, Mortimer J, Estes N, Haller DG, Ajani J, Kocha W, Minsky BD, Roth JA (1998). Chemotherapy followed by surgery compared with surgery alone for localized esophageal cancer. N Engl J Med.

[4] Bhansali MS, Fujita H, Kakegawa T, Yamana H, Ono T, Hikita S, Toh Y, Fujii T, Tou U, Shirouzu K (1997). Pattern of recurrence after extended radical esophagectomy with three-field lymph node dissection for squamous cell carcinoma in the thoracic esophagus. World J Surg.

[5] Kyriazanos ID, Tachibana M, Shibakita M, Yoshimura H, Kinugasa S, Dhar DK, Nakamoto T, Fujii T, Nagasue N (2003). Pattern of recurrence after extended esophagectomy for squamous cell carcinoma of the esophagus. Hepatogastroenterology.

[6] Liu P, Cheng H, Roberts TM, Zhao JJ (2009). Targeting the phosphoinositide 3-kinase pathway in cancer. Nat Rev Drug Discov.

[7] Engelman JA (2009). Targeting PI3K signalling in cancer: opportunities, challenges and limitations. Nat Rev Cancer.

[8] Chappell WH, Steelman LS, Long JM, Kempf RC, Abrams SL, Franklin RA, Basecke J, Stivala F, Donia M, Fagone P, Malaponte G, Mazzarino MC, Nicoletti F (2011). Ras/Raf/MEK/ERK and PI3K/PTEN/Akt/mTOR inhibitors: rationale and importance to inhibiting these pathways in human health. Oncotarget.

[9] McCubrey JA, Steelman LS, Chappell WH, Sun L, Davis NM, Abrams SL, Franklin RA, Cocco L, Evangelisti C, Chiarini F, Martelli AM, Libra M, Candido S (2012). Advances in targeting signal transduction pathways. Oncotarget.

[10] Li B, Tsao SW, Li YY, Wang X, Ling MT, Wong YC, He QY, Cheung AL (2009). Id-1 promotes tumorigenicity and metastasis of human esophageal cancer cells through activation of PI3K/AKT signaling pathway. Int J Cancer.

[11] Li B, Tsao SW, Chan KW, Ludwig DL, Novosyadlyy R, Li YY, He QY, Cheung AL (2014). Id1-Induced IGF-II and Its Autocrine/Endocrine Promotion of Esophageal Cancer Progression and Chemoresistance--Implications for IGF-II and IGF-IR-Targeted Therapy. Clin Cancer Res.

[12] Bartholomeusz C, Gonzalez-Angulo AM (2012). Targeting the PI3K signaling pathway in cancer therapy. Expert Opin Ther Targets.

[13] Hildebrandt MA, Yang H, Hung MC, Izzo JG, Huang M, Lin J, Ajani JA, Wu X (2009). Genetic variations in the PI3K/PTEN/AKT/mTOR pathway are associated with clinical outcomes in esophageal cancer patients treated with chemoradiotherapy. J Clin Oncol.

[14] Li B, Cheung PY, Wang X, Tsao SW, Ling MT, Wong YC, Cheung AL (2007). Id-1 activation of PI3K/Akt/NFkappaB signaling pathway and its significance in promoting survival of esophageal cancer cells. Carcinogenesis.

[15] Sourbier C, Lindner V, Lang H, Agouni A, Schordan E, Danilin S, Rothhut S, Jacqmin D, Helwig JJ, Massfelder T (2006). The phosphoinositide 3-kinase/Akt pathway: a new target in human renal cell carcinoma therapy. Cancer Res.

[16] Zito CR, Jilaveanu LB, Anagnostou V, Rimm D, Bepler G, Maira SM, Hackl W, Camp R, Kluger HM, Chao HH (2012). Multi-level targeting of the phosphatidylinositol-3-kinase pathway in non-small cell lung cancer cells. PLoS One.

[17] Mallon R, Feldberg LR, Lucas J, Chaudhary I, Dehnhardt C, Santos ED, Chen Z, dos SO, Ayral-Kaloustian S, Venkatesan A, Hollander I (2011). Antitumor efficacy of PKI-587, a highly potent dual PI3K/mTOR kinase inhibitor. Clin Cancer Res.

[18] Weber GL, Parat MO, Binder ZA, Gallia GL, Riggins GJ (2011). Abrogation of PIK3CA or PIK3R1 reduces proliferation, migration, and invasion in glioblastoma multiforme cells. Oncotarget.

[19] Opel D, Naumann I, Schneider M, Bertele D, Debatin KM, Fulda S (2011). Targeting aberrant PI3K/Akt activation by PI103 restores sensitivity to TRAIL-induced apoptosis in neuroblastoma. Clin Cancer Res.

[20] Hong DS, Bowles DW, Falchook GS, Messersmith WA, George GC, O'Bryant CL, Vo AC, Klucher K, Herbst RS, Eckhardt SG, Peterson S, Hausman DF, Kurzrock R (2012). A multicenter phase I trial of PX-866, an oral irreversible phosphatidylinositol 3-kinase inhibitor, in patients with advanced solid tumors. Clin Cancer Res.

[21] Janku F, Wheler JJ, Westin SN, Moulder SL, Naing A, Tsimberidou AM, Fu S, Falchook GS, Hong DS, Garrido-Laguna I, Luthra R, Lee JJ, Lu KH (2012). PI3K/AKT/mTOR inhibitors in patients with breast and gynecologic malignancies harboring PIK3CA mutations. J Clin Oncol.

[22] Zhou YA, Zhang T, Zhao JB, Wang XP, Jiang T, Gu ZP, Wang XN, Li XF (2010). The adenovirus-mediated transfer of PTEN inhibits the growth of esophageal cancer cells *in vitro* and *in vivo*. Biosci Biotechnol Biochem.

[23] Goncharenko-Khaider N, Lane D, Matte I, Rancourt C, Piche A (2010). The inhibition of Bid expression by Akt leads to resistance to TRAIL-induced apoptosis in ovarian cancer cells. Oncogene.

[24] Simioni C, Martelli AM, Cani A, Cetin-Atalay R, McCubrey JA, Capitani S, Neri LM (2013). The AKT inhibitor MK-2206 is cytotoxic in hepatocarcinoma cells displaying hyperphosphorylated AKT-1 and synergizes with conventional chemotherapy. Oncotarget.

[25] Yoshioka A, Miyata H, Doki Y, Yasuda T, Yamasaki M, Motoori M, Okada K, Matsuyama J, Makari Y, Sohma I, Takiguchi S, Fujiwara Y, Monden M (2008). The activation of Akt during preoperative chemotherapy for esophageal cancer correlates with poor prognosis. Oncol Rep.

[26] McCubrey JA, Steelman LS, Chappell WH, Abrams SL, Montalto G, Cervello M, Nicoletti F, Fagone P, Malaponte G, Mazzarino MC, Candido S, Libra M, Basecke J, Mijatovic S (2012). Mutations and deregulation of Ras/Raf/MEK/ERK and PI3K/PTEN/Akt/mTOR cascades which alter therapy response. Oncotarget.

[27] Shimada Y, Imamura M, Wagata T, Yamaguchi N, Tobe T (1992). Characterization of 21 newly established esophageal cancer cell lines. Cancer.

[28] Hu Y, Lam KY, Wan TS, Fang W, Ma ES, Chan LC, Srivastava G (2000). Establishment and characterization of HKESC-1, a new cancer cell line from human esophageal squamous cell carcinoma. Cancer Genet Cytogenet.

[29] Kawamata H, Furihata T, Omotehara F, Sakai T, Horiuchi H, Shinagawa Y, Imura J, Ohkura Y, Tachibana M, Kubota K, Terano A, Fujimori T (2003). Identification of genes differentially expressed in a newly isolated human metastasizing esophageal cancer cell line, T. Tn-AT1, by cDNA microarray. Cancer Sci.

[30] Li B, Li YY, Tsao SW, Cheung AL (2009). Targeting NF-kappaB signaling pathway suppresses tumor growth, angiogenesis, and metastasis of human esophageal cancer. Mol Cancer Ther.

